# Ultrafiltration Rate Thresholds Associated With Increased Mortality Risk in Hemodialysis, Unscaled or Scaled to Body Size

**DOI:** 10.1016/j.ekir.2022.04.079

**Published:** 2022-04-22

**Authors:** Jochen G. Raimann, Yuedong Wang, Ariella Mermelstein, Peter Kotanko, John T. Daugirdas

**Affiliations:** 1 Renal Research Institute, New York, New York, USA; 2 Department of Statistics and Applied Probability, University of California, Santa Barbara, Santa Barbara, California, USA; 3 Department of Medicine, Icahn School of Medicine at Mount Sinai, New York, New York, USA; 4 Department of Medicine, University of Illinois at Chicago, Chicago, Illinois, USA

**Keywords:** hemodialysis, mortality, outcome, ultrafiltration

## Abstract

**Introduction:**

One proposed threshold ultrafiltration rate (UFR) of concern in hemodialysis patients is 13 ml/h per kg. We evaluated associations among UFR, postdialysis weight, and mortality to determine whether exceeding such a threshold would result in similar levels of risk for patients of different body weights.

**Methods:**

Data were analyzed in this retrospective cohort study for 1 year following dialysis initiation (baseline) and over 2 years of follow-up in incident patients receiving thrice-weekly in-center hemodialysis. Patient-level UFR was averaged over the baseline period. To investigate the joint effect of UFR and postdialysis weight on survival, we fit Cox proportional hazards models using bivariate tensor product spline functions, adjusting for sex, race, age, diabetes, and predialysis serum albumin, phosphorus, and systolic blood pressure (BP). We constructed contour plots of mortality hazard ratios (MHRs) over the entire range of UFR values and postdialysis weights.

**Results:**

In the studied 2542 patients, UFR not scaled to body weight was strongly associated with MHR, whereas postdialysis weight was inversely associated with MHR. MHR crossed 1.5 when unscaled UFR exceeded 1000 ml/h, and this relationship was largely independent of postdialysis weight in the range of 80 to 140 kg. A UFR warning level associated with a lower MHR of 1.3 would be 900 ml/h, whereas the UFR associated with an MHR of 1.0 was patient-size dependent. The MHR when exceeding a UFR threshold of 13 ml/h per kg was dependent on patient weight (MHR = 1.20, 1.45, and >2.0 for a 60, 80, and 100 kg patient, respectively).

**Conclusion:**

UFR thresholds based on unscaled UFR give more uniform risk levels for patients of different sizes than thresholds based on UFR/kg.

## Graphical abstract

**Figure:**
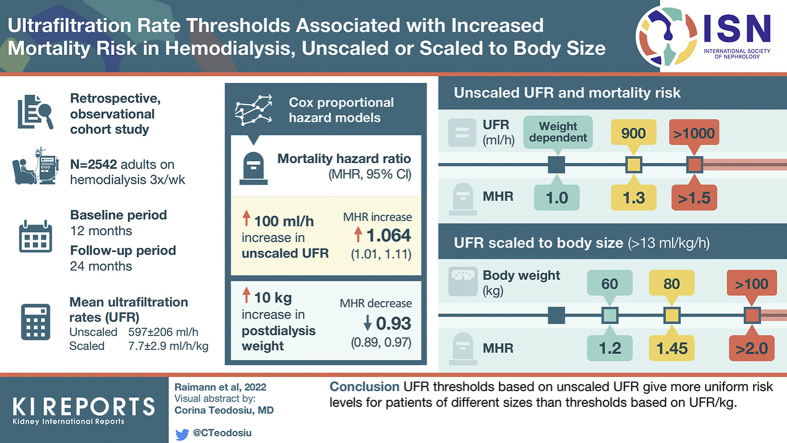

See Commentary on Page 1456

On the basis of some early work that identified an association between UFR scaled to body weight and mortality,^1^, ^2^, ^3^ it has become commonplace to use UFR/kg as a quality assurance measure, with a recommendation to limit UFR to less than 13 ml/h per kg. For example, the number of patients remaining below this level of UFR/kg has been set by the Centers for Medicare and Medicaid Services in the United States as a quality assurance metric. More recent observational data in very large patient populations^4^, ^5^, ^6^ seemed to confirm the rationale for such an approach. However, the reason for scaling UFR to body weight in these initial studies was never fully expressed. One potential problem with scaling UFR to body weight is the strong inverse association between body weight and adverse outcomes in dialysis patients,^7^^,^^8^ with mortality being markedly increased in patients of low body weight. The reasons for the inverse association are not completely known. Presumably some of the association may be due to better nutritional status of larger patients, a lower ratio of visceral-to-lean body mass, and/or increased resistance to the effects of uremia.^9^, ^10^, ^11^ UFR divided by body weight might not be associated with a uniform increase in MHR for different levels of body size, as suggested by preliminary data published in abstract form by Larkin *et al.*^12^ In reanalyzing a recently published data set of the association of UFR/kg with MHR by strata of body weight,^6^ one of us (JTD) pointed out that a UFR limit of 13 ml/h per kg was exceeded in larger patients at a greater MHR than when the limit was exceeded in smaller patients.^13^ A better approach might be to evaluate the joint effects of unscaled UFR and postdialysis weight on mortality. Here, we present the results of such an analysis in a sample of hemodialysis patients.

## Methods

### Study Design

This retrospective, observational cohort study was conducted in patients commencing thrice-weekly maintenance HD in clinics of the Renal Research Institute between January 1, 2014, and October 31, 2018. Data from electronic health records that captured all clinical and treatment-related data from patients receiving treatment were extracted in a deidentified format. Patients >18 years receiving thrice-weekly hemodialysis who survived at least 12 months were included in this analysis. From the extracted data set, we defined the first 12 months as the baseline period and the following 2 years as the follow-up period. The Western Institutional Review Board deemed the study exempt from human subject consent requirements and continuing review.

### Measurements

For the primary analysis, baseline parameters were determined as the mean of all available entries during the first 12 months of dialysis. Missing data were excluded from the computation of the mean and the SD. For a subsequent sensitivity analysis, we restricted the baseline to months 11 and 12 during the first year of renal replacement therapy. The UFR was calculated as the predialysis minus postdialysis weight difference divided by dialysis session length. The UFR scaled to weight was calculated as the UFR divided by the postdialysis body weight. Interdialytic weight gain (IDWG) was calculated as the mean of every predialysis weight minus the preceding postdialysis weight. All parameters were routinely collected in the electronic health records and extracted for the purpose of analysis. Race data were based on patient self-identification as recorded in the Fresenius North America and Renal Research Institute patient database. The reason for reporting race data was to more completely characterize the study population.

### Statistical Analysis

Data are reported as mean ± SD unless stated otherwise. Pearson correlation was used to evaluate association between variables. Over the 2-year follow-up period, all-cause mortality was assessed from the electronic health record. We were mainly interested in the associations between unscaled UFR and scaled UFR and all-cause death during the 2-year follow-up period. We fit Cox proportional hazard models with unscaled or scaled UFR and postdialysis weight as continuous independent variables, adjusting for sex, race, age, and diabetic status. As a sensitivity analysis, additional adjustments were made for predialysis systolic BP, serum albumin, and serum phosphate. To explore potential nonlinear effects and interactions between unscaled or scaled UFR and postdialysis weight, we used a bivariate tensor product spline function of UFR and postdialysis weight.^14^ The bivariate spline function estimates the joint effect of UFR and postdialysis weight without assuming any specific form of the bivariate function. The bivariate function explores both the main effects of and interaction between UFR and postdialysis weight. To construct a continuous version of relative mortality risk, we first fit a linear model with the postdialysis weight as the independent variable and unscaled or scaled UFR as the dependent variable. We computed MHRs on grid points of postdialysis weight and UFR and then constructed contour plots. A line on the contour plots represents values of postdialysis weight and UFR for which MHR equals a constant marked on the line. For each fixed postdialysis weight, we used the expected UFR at this postdialysis weight as the reference point and computed MHRs at different UFRs. As a sensitivity analysis aiming to evaluate a potential biases, we restricted the baseline period to the last 2 months of the first year and repeated the outcome analysis with the resulting aggregated variables as the predictors. Further sensitivity analyses conducted used the same methodologic approach replacing UFR by (i) UFR normalized to postdialysis weight, (ii) scaled to body surface area (BSA) according to the Dubois equation,^15^ and (iii) UFR scaled to postdialysis weight raised to the power of 0.4.

The statistical software R 4.1.2, codename “Bird Hippie” with packages *dplyr*, *tidyr*, *survival*, *mgcv*, *survminer*, and *doBy*, was used for all analyses.^16^ A *P* < 5% was considered significant.

## Results

The demographics and basic clinical characteristics of the 2542 patients studied were as follows: mean (± SD) age was 61.9 ± 15 years, 42% were female, 40% African American, and 38.8% had evidence or history of diabetes mellitus. The postdialysis weight averaged 81.0 ± 23.5 kg. The mean unscaled UFR was 597 ± 206 ml/h, and the mean scaled UFR was 7.7 ± 2.9 ml/h per kg. Baseline data are given in Table 1 and a flow diagram of patient recruitment is found in Supplementary Figure S1. The data presented in Table 1 are averages of all values present in the electronic medical record during the baseline year 1. A histogram of the counts of UFR, BP, and laboratory values during year 1 from which the averages were determined is found in Supplementary Figure S2.

**Table 1:** Patient characteristics

Number of patients	2542
Sex	
Male, count (%)	1466 (57.7)
Height (cm)	168.5 ± 10.8
Age	61.9 ± 15.0
Diabetes (%)	38.8
Race (self-identified), count (%)	
White	1075 (42.3)
Black	1025 (40.4)
Unknown	348 (13.7)
Asian	67 (2.6)
Native Hawaiian/Other Pacific Islander	25 (1.0)
American Indian or Alaskan Native	2 (0.08)
Treatment and laboratory values, mean ± SD	
Ultrafiltration rate (ml/h)	597 ± 206
Scaled ultrafiltration rate (ml/h per kg)	7.7 ± 2.9
Session length (min)	220 ± 33
Kt/V	1.64 ± 0.24
URR	74.5 ± 4.88
Predialysis pulse pressure (mm Hg)	71 ± 14.2
Predialysis systolic BP (mm Hg)	149.9 ± 18.02
Predialysis serum albumin (g/dl)	3.82 ± 0.32
Predialysis serum phosphorus (mg/dl)	5.43 ± 1.15
Interdialytic weight gain (kg)	2.18 ± 0.89
Predialysis weight (kg)	83.2 ± 23.9
Postdialysis weight (kg)	81.0 ± 23.4
Body mass index (kg/m^2^)	28.5 ± 7.58

BP, blood pressure; URR, urea reduction ratio expressed as percent.

Treatment, BP, and laboratory values listed are based on patient average values calculated from all data available in the electronic medical record during baseline year 1.

The frequency distributions for 12-month averaged UFR and postdialysis weight are found in Supplementary Figure S3A and B. The unscaled UFR was associated with postdialysis body weight, (r = 0.31; Supplementary Figure S4A), whereas UFR/kg was inversely associated with postdialysis weight (r = –0.45; Supplementary Figure S4B). Because some investigators adjusted mortality analyses versus UFR/kg for IDWG, whereas others did not, claiming collinearity, we evaluated the association of UFR with IDWG. In our data, we found collinearity between these 2 variables, as indicated by a significant correlation (r = 0.91).

**Figure 1:**
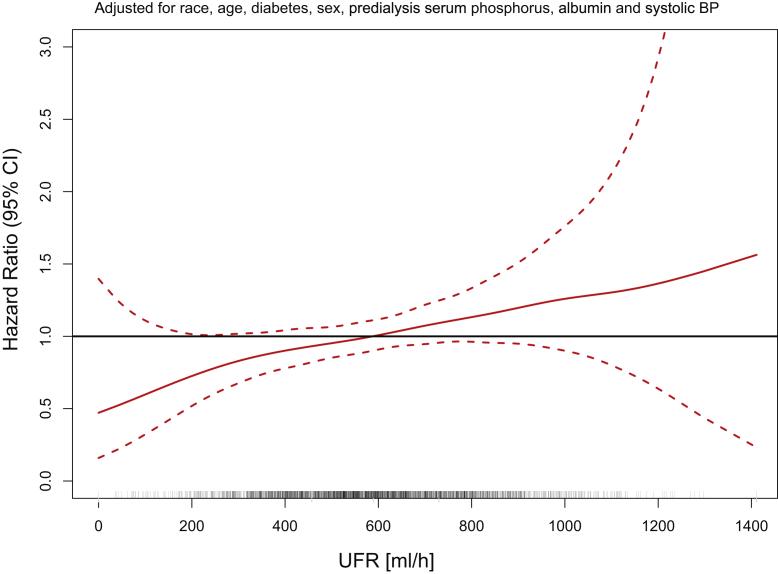
Association between unscaled UFR and mortality hazard ratio. The analysis was adjusted for age, sex, diabetes, race, postdialysis weight, predialysis serum albumin, phosphorus, and systolic BP. BP, blood pressure; UFR, ultrafiltration rate.

**Figure 2:**
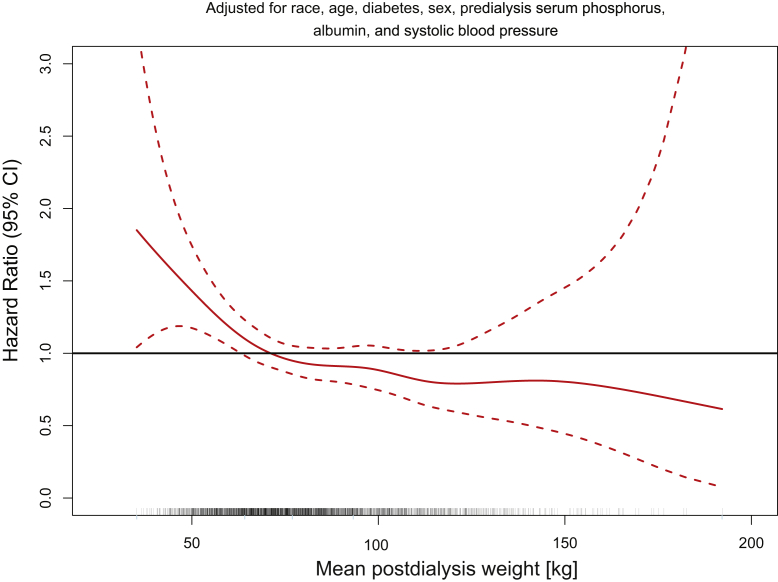
Association between postdialysis weight and mortality hazard ratio. The analysis was adjusted for age, sex, diabetes, race, predialysis serum albumin, phosphorus, and systolic blood pressure.

During the 2-year follow-up period (totaling 3479 patient-years during the first year of follow-up and 1393 patient-years during the second year) 494 patients (270 during the first and 224 during the second year of follow-up) died. The resulting mortality rates for the first and the second years of follow-up were 7.8 and 16.1 deaths per 100 patient-years. Figures 1 and 2 reveal the spline estimates of the MHR functions with unscaled UFR and postdialysis weight as independent variables, respectively, adjusted for age, sex, diabetes, race, predialysis serum albumin, phosphorus, and systolic BP. Because these spline estimates were close to linear, we refit Cox proportional hazard models with linear functions. On the basis of these linear Cox proportional hazard models, an increase in UFR of 100 ml/h was associated with an increase in MHR of 1.064 (95% CI: 1.010–1.114, *P* = 0.0121), whereas a postdialysis weight increment of 10 kg was associated with a decreased MHR of 0.933 (95% CI: 0.891–0.979, *P* = 0.0049).

Because of the complex nonlinear inter-relationships between unscaled UFR or UFR/kg and postdialysis weight, and possibly independent associations of mortality with UFR measures and weight, we elected to evaluate the relationships among UFR (unscaled or scaled to body weight), weight, and mortality, using 2-dimensional contour plots where weight was on 1 dimension, and UFR, unscaled or scaled to various body size metrics, on the other. These analyses were controlled for age, sex, diabetes, and race and for predialysis serum albumin, phosphorus, and systolic BP. Supplementary Figure S5 illustrates slice plots revealing estimated MHR versus unscaled UFR for various levels of body weight, based on this contour plot analysis.

**Figure 3:**
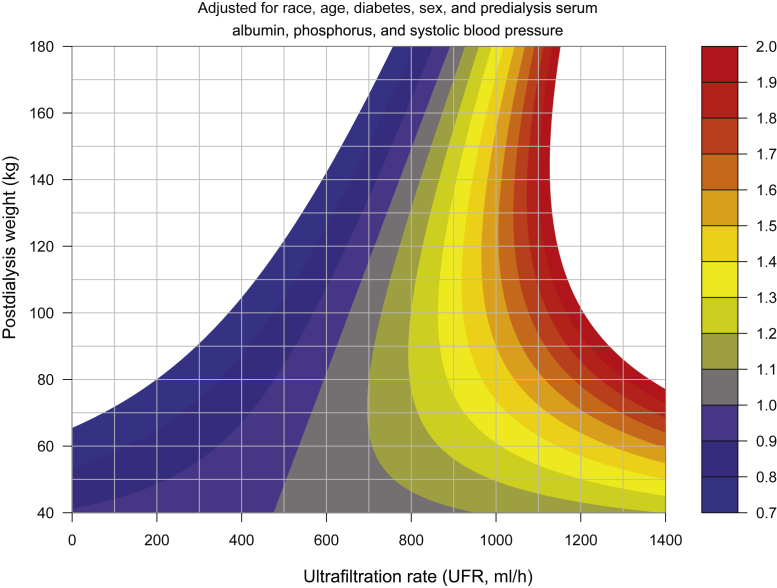
Unscaled UFR: Contour plots of MHRs as a function of postdialysis weight on the vertical axis, and unscaled UFR on the horizontal axis. The color scheme indicates discrete levels of MHRs. The analysis was adjusted for age, sex, diabetes, race, predialysis serum albumin, phosphorus, and systolic blood pressure. MHR, mortality hazard ratio; UFR, ultrafiltration rate.

**Figure 4:**
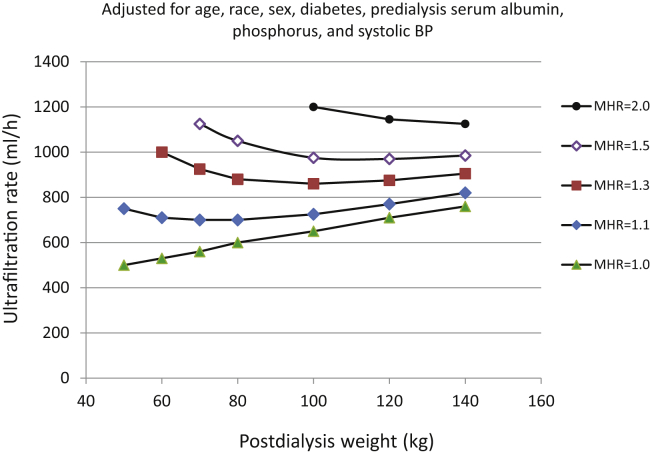
Plots of MHR at different levels of risk by weight, with data taken from Figure 5. BP, blood pressure; MHR, mortality hazard ratio.

Figure 3 illustrates contour plots for unscaled UFR (ml/h). In the contour plots, the isopleths represent areas of equal MHR. An MHR of 1.0 represents average mortality risk for a particular weight stratum. For all estimates of UFR scaling, the spacing between MHR isopleths progressively increased at lower body weights, becoming especially wide when postdialysis weight was <60 kg. Figure 4 illustrates MHR levels of 1.1, 1, 3, 1.5, and 2.0 for different body weights. The data are taken from the contour plot in Figure 3. Supplementary Table S1 illustrates estimated MHR levels at various levels of unscaled UFR using either the average UFR of the entire year 1 baseline period versus an average UFR computed from values during months 11 and 12 of the baseline period.

**Figure 5:**
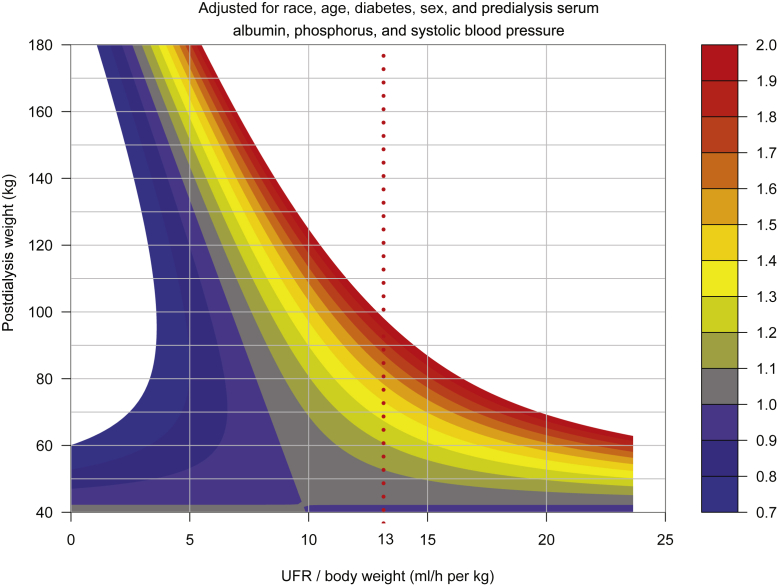
UFR/kg: Contour plots of MHRs as a function of postdialysis weight on the vertical axis, and UFR scaled to postdialysis weight on the horizontal axis. The color scheme indicates discrete levels of MHRs. The analysis was adjusted for age, sex, diabetes, race, predialysis serum albumin, phosphorus, and systolic blood pressure. The vertical dashed line marks a UFR rate of 13 ml/h per kg. MHR, mortality hazard ratio; UFR, ultrafiltration rate.

**Figure 6:**
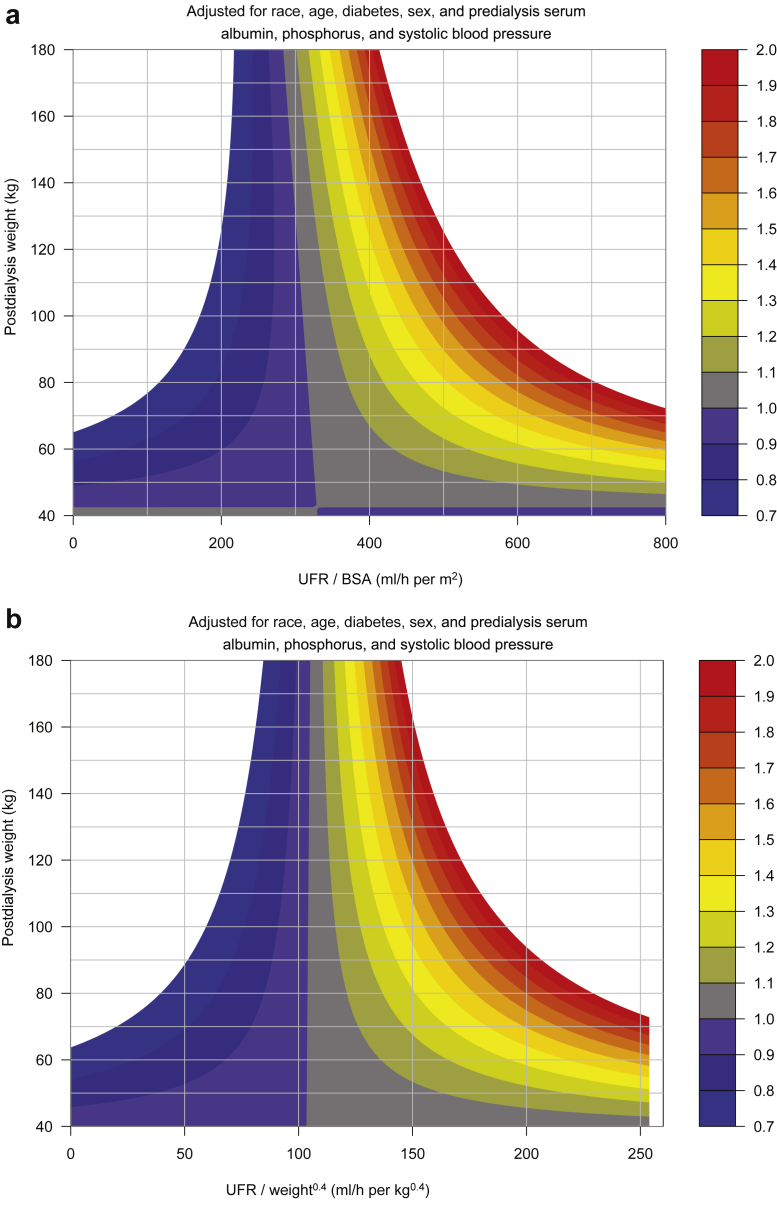
(a) UFR/m^2^: Contour plots of mortality hazard ratios (MHRs) as a function of postdialysis weight on the vertical axis, and UFR scaled to BSA (calculated using the Dubois equation) on the horizontal axis. (b) UFR/kg^0.4^: Contour plots of MHRs as a function of postdialysis weight on the vertical axis, and UFR scaled to postdialysis weight to the 0.4 power (UFR/kg^0.4^) on the horizontal axis. The color scheme indicates discrete levels of MHRs. The analyses were adjusted for age, sex, diabetes, race, predialysis serum albumin, phosphorus, and systolic blood pressure. BSA, body surface area; MHR, mortality hazard ratio; UFR, ultrafiltration rate.

In Figure 5, the UFR metric is UFR scaled to postdialysis weight (ml/h per kg). In Figure 6a, the UFR is scaled to BSA calculated by the Dubois equation.^15^ Figure 6b reveals results with UFR scaled to postdialysis weight raised to the 0.4 power (UFR/kg^0.4^). Supplementary Figure S6 illustrates a scatterplot of estimated blood volume against BSA.

For unscaled UFR (Figure 3), the MHR = 1.0 isopleth sloped to the right, whereas slopes for MHR isopleths denoting increasing risk were closer to vertical. For UFR/kg, the MHR = 1.0 isopleth sloped markedly to the left, as did the isopleths for higher MHR values (Figure 5). The MHR = 1.0 isopleth was close to vertical for UFR/m^2^ but still sloped to the left (Figure 6a), and the MHR=1.0 isopleth was also near vertical for UFR scaled to weight^0.4^ (Figure 6b).

## Discussion

Our results confirm the adverse association between UFR and mortality suggested by previous investigators. This is the first published article to our knowledge which evaluated the association of mortality and unscaled UFR; previous analyses looked at the associations of mortality with UFR scaled to body weight (UFR/kg).^1^, ^2^, ^3^, ^4^, ^5^, ^6^ We believe that analyses of mortality versus UFR/kg are valid; however, our data suggest that UFR/kg warning thresholds are problematic, as the MHR associated with a given UFR/kg level is strongly dependent on body weight. When the currently popular UFR/kg threshold of 13 ml/h per kg is exceeded, our data suggest that the MHR is substantially greater in larger patients (Figure 5). This potential issue was, in fact, noted to be present^13^ in some previous analyses done by others.

Trying to determine the causal effect of UFR on mortality by looking at observational data is difficult. UFR is confounded with many volume-related factors that might also affect survival. Mathematically, average UFR on a weekly time scale can be calculated as weekly fluid intake minus weekly urine output and insensible fluid loss divided by weekly dialysis session length. Fluid intake minus fluid loss plus insensible loss will determine IDWG. Of 5 previous studies evaluating the association of UFR/kg and mortality, 3 adjusted the UFR/kg versus mortality analysis for IDWG. Two investigators^4^^,^^6^ did not, citing the collinearity of UFR and IDWG.^4^ We were reticent to adjust our analyses for IDWG because we found the IDWG and UFR to indeed be highly correlated (adjusted r value of 0.91; Supplementary Figure S7). The IDWG and UFR variables are not only collinear in an arithmetic sense, but also causally related.

The relationship between IDWG and mortality has been extensively studied by others, and there are many factors to consider: for example, a high degree of residual renal function and urine output will lower IDWG and UFR. Thus, a low IDWG or UFR might be a marker for residual renal function, and there may be some amount of survival benefit by this mechanism. In contrast, a higher IDWG or UFR may signify a patient who is taking in more sodium, which is related to caloric intake; higher food intake might be associated with better patient health. In our patient data set, postdialysis weight was closely associated with body mass index, and most smaller patients had a low value of body mass index (Supplementary Figure S8). It is likely that small, low body mass index patients tend to be malnourished, and a lower UFR, in those in whom IDWG is also low, might represent patients who are not eating because of chronic illness and who may have a poor outcome for this reason.

Our data confirmed the association between low postdialysis weight and poor outcome that has been described by many previous studies. The reasons why survival in lighter dialysis patients is poorer than in their heavier counterparts are complex, and detailed studies of body components which determine weight and its effects on outcome have been done.^9^, ^10^, ^11^ It is problematic, in our opinion, to attempt to determine the effects of UFR on survival by scaling UFR to body weight, given that high UFR/kg values may be due either to a high UFR value or alternatively due to a low value for the denominator of the UFR/kg term, namely, body weight. The inverse association between body weight and mortality is of substantial magnitude, and causally, the low body weight factors associated with poor survival are unlikely to be related to volume overload or to dialysis hypotension or dialysis-treatment-related complications.

Our contour plots suggest that to achieve an MHR of 1.0 for patients of various body size, a UFR scaling factor of weight^0.4^ or UFR/BSAs (BSA) would be preferable to either unscaled UFR or UFR/kg. With unscaled UFR, the neutral MHR (MHR = 1.0) isopleth sloped to the right, whereas for UFR/kg, the neutral MHR isopleth sloped markedly to the left. A physiological argument for scaling UFR to BSA was proposed by Daugirdas and Schneditz.^17^ This was based on the fact that blood volume in overweight humans scales more closely to BSA than body weight,^18^ although this blood volume scaling hypothesis has not yet been rigorously tested. The neutral risk (MHR = 1.0) isopleth when UFR was scaled as UFR/m^2^ is found in Figure 6a. Its slope slants slightly to the right. In the Dubois estimating equation, BSA = 0.007814 × weight-kg^0.425^ × height-cm^0.725^. We did create MHR contour plots for UFR scaled to body weight to the 0.4 power (Figure 6b). In these plots, the neutral MHR = 1.0 isopleths for UFR/kg^0.4^ were also very close to vertical. The Lemmens estimate of blood volume and Dubois surface area were very tightly correlated (Supplementary Figure S6).

Our study has several strengths and some limitations. As with all observational studies, residual confounding may remain in this analysis. The UFR and body weight were averaged values over a 12-month period, helping ensure that the values calculated would be good estimates of the actual UFR characteristics of each patient. Although substantial, the number of patients followed may not have been large enough to provide adequate generalizability. Furthermore, results may also differ with different follow-up times. We chose to evaluate the association with a follow-up 2 years. It is possible that in analyses of larger patient data sets studied over a longer follow-up period that values of UFR associated with various mortality risk thresholds may be different.

Another caveat and limitation regarding these UFR warning levels has to do with patient selection. The patients in our study were all being dialyzed in the United States. Usvyat *et al.*^19^ have revealed that average IDWGs and session lengths differ among patients being dialyzed in the Asia Pacific region and Europe compared with those dialyzed in North America. Accordingly, it is possible that the relative mortality risks associated with specific UFR levels in Europe and the Asia Pacific countries differ from relative risk in patients dialyzed in North America. There was a trend in our data suggesting that associations among unscaled ultrafiltration rate, body weight, and mortality hazard ratio might differ between men and women, but the sex effect was not statistically significant in the Cox regression analysis. It would be useful to explore any potential sex-related differences in a larger patient dataset.

We studied UFR as a long-term exposure variable, studied as an average value. It is possible that additional risk might be related with variability of the UFR. Especially, episodic high values might be associated with intradialytic injury to the cardiovascular system and with a worsened outcome. The possible association between intrapatient UFR variability and outcome is an important subject for further study and analysis.

Our data suggest that for United States dialysis patients, a UFR warning threshold in the range of 900 ml/h could be a candidate warning level for patients with postdialysis weight range of 80 to 140 kg, as exceeding such a UFR was associated with a MHR of approximately 1.3, largely unrelated to patient size. Our data suggest that exceeding a UFR of 1000 ml/h is associated with an MHR of 1.5, even for patients who are quite large. UFR associated with lesser levels of risk might be chosen based on some multiple of the MHR = 1.0 isopleth shown in Figures 3 or 6b. For lower levels of MHR, there is a slight association of UFR warning level with body weight: UFR levels associated with an MHR of 1.0, for example, would be 500 to 600 ml/h for patients weighing ≤80 kg, and 600 to 750 ml/h for patients weighing 80 to 120 kg. Our contour plot analysis does suggest that the currently accepted UFR risk threshold of 13 ml/kg per hour may disadvantage larger patients and thresholds based on unscaled UFR may be preferable. More work is needed to determine the mortality risks associated with higher UFRs in smaller patients. Our data do suggest that thresholds of unscaled UFR are associated with more uniform levels of risk for patients of different body sizes than thresholds of UFR scaled to body weight.

## Disclosure

JGR, AM, and PK are employees of the Renal Research Institute, a wholly owned subsidiary of Fresenius Medical Care. PK holds stock in Fresenius Medical Care and receives author honoraria from Henry Stewart Talks. All the other authors declared no competing interests.

## Supplementary Material

Supplementary File (PDF)

**Figure S1.** Flow diagram of patient recruitment.

**Figure S2.** Histograms of number of counts from which averages were determined during baseline year 1.

**Figure S3.** (a) Frequency distribution of mean unscaled ultrafiltration rate during the 12-month baseline period (ml/h). (b) Frequency distribution of mean postdialysis weight during the 12-month baseline period (kg).

**Figure S4.** (a) Scatterplot of unscaled UFR against postdialysis weight, r = 0.34. (b) Scatterplot of UFR/kg against postdialysis weight, r = 0.45.

**Figure S5.** Slice plots revealing estimated mortality hazard ratio versus ultrafiltration rate for various levels of body weight, based on contour plot analysis.

**Figure S6.** Scatterplot of estimated blood volume calculated using the Lemmens equation (ref. 17) against body surface area.

**Figure S7.** Scatterplot of average interdialytic weight gain (IDWG) versus average ultrafiltration rate (UFR) during the baseline period.

**Figure S8.** Scatterplot of postdialysis body weight versus body mass index.

**Table S1.** Mortality hazard ratios versus unscaled ultrafiltration rate (UFR) using the described contour plot analysis, comparing use of a 12-month year 1 average UFR versus a 2-month average UFR (from months 11 and 12 of the baseline year 1).

**STROBE statement.**
