# Supplementary material for: Ultrafiltration Rate Thresholds Associated With Increased Mortality Risk in Hemodialysis, Unscaled or Scaled to Body Size
Source: Kidney Int Rep. 2022 Apr 22;7(7):1585–93. doi: 10.1016/j.ekir.2022.04.079 (PMC9263411; doi:10.1016/j.ekir.2022.04.079)
Supplement: Supplementary File (PDF) [file mmc1.pdf]

Raimann et al. 07 Apr 2022

Ultrafiltration rate thresholds associated with increased mortality risk in hemodialysis, unscaled or scaled to body size

Jochen G. Raimann[1] (ORCID ID 0000-0002-8954-2783), Yuedong Wang[2], Ariella Mermelstein[1], Peter G. Kotanko[1,3], and John T. Daugirdas[4]

SUPPLEMENTAL DATA CONTENTS:

Supplemental data Figure S1.
Flow diagram of patient recruitment

Supplemental data Figure S2.
Histograms of number of counts from which averages were determined during baseline year 1.

Supplemental data Figure S3a.
Frequency distribution of mean unscaled ultrafiltration rate during the 12-month baseline period (ml/hr).

Supplemental data Figure S3b.
Frequency distribution of mean postdialysis weight during the 12-month baseline period (kg).

Supplemental data Figure S4a.
Scatterplot of unscaled UFR against postdialysis weight, r = 0.34.

Supplemental data Figure S4b.
Scatterplot of UFR/kg against postdialysis weight, r = 0.45.

Supplemental data Figure S5. Slice plots showing estimated mortality hazard ratio vs. ultrafiltration rate for various levels of body weight, based on contour plot analysis.

Supplemental data Figure S6.
Scatterplot of estimated blood volume calculated using the Lemmens equation (ref. 17) against body surface area.

Supplemental data Figure S7;
Scatterplot of average interdialytic weight gain (IDWG) vs. average ultrafiltration rate (UFR) during the baseline period.

Supplemental data Figure S8.
Scatterplot of postdialysis body weight vs. body mass index.

Supplemental data Table S1. Comparison of mortality hazard ratios with UF rate averaged during months 1–12 of year 1 vs. months 11–12 of year 1.

STROBE Statement.

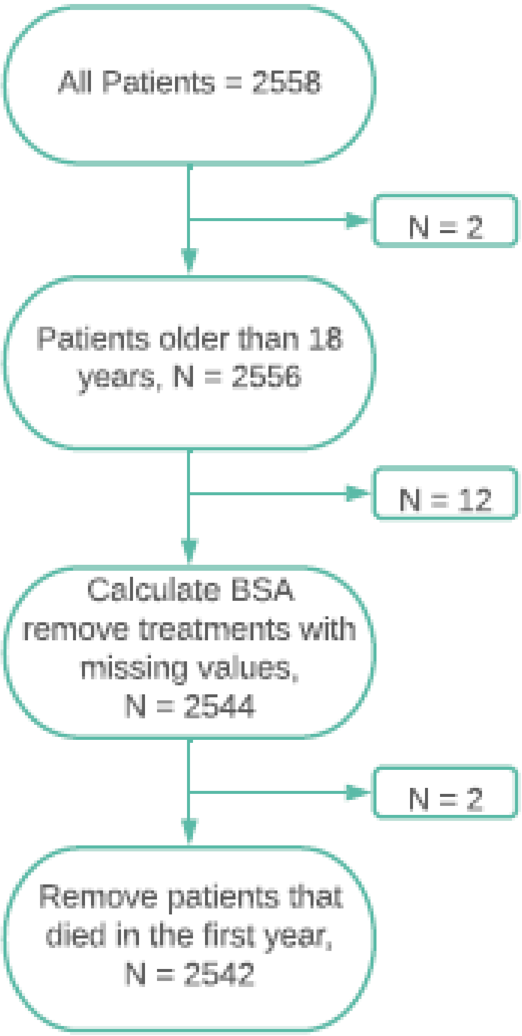

Supplemental data Figure S1.
Flow diagram of patient recruitment

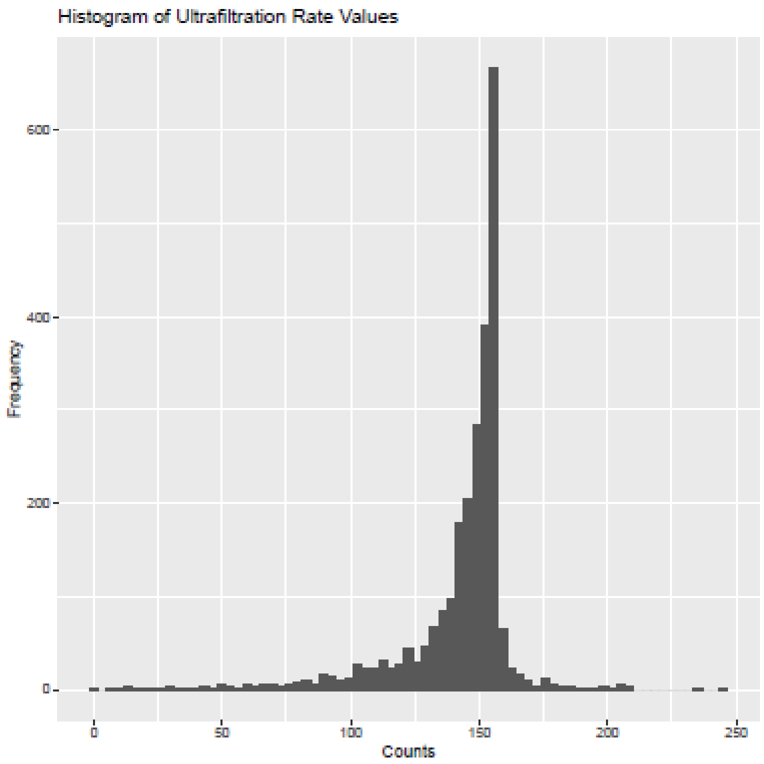
Histogram of Ultrafiltration Rate Values
Frequency
Counts
0
200
400
600
0
50
100
150
200
250

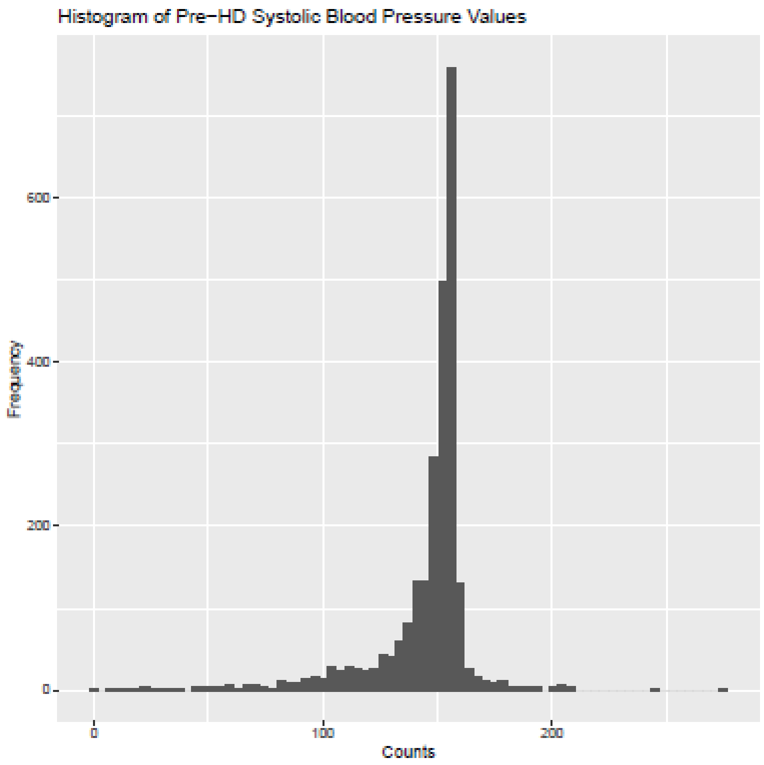
Histogram of Pre−HD Systolic Blood Pressure Values
Frequency
Counts
0
200
400
600
0
100
200

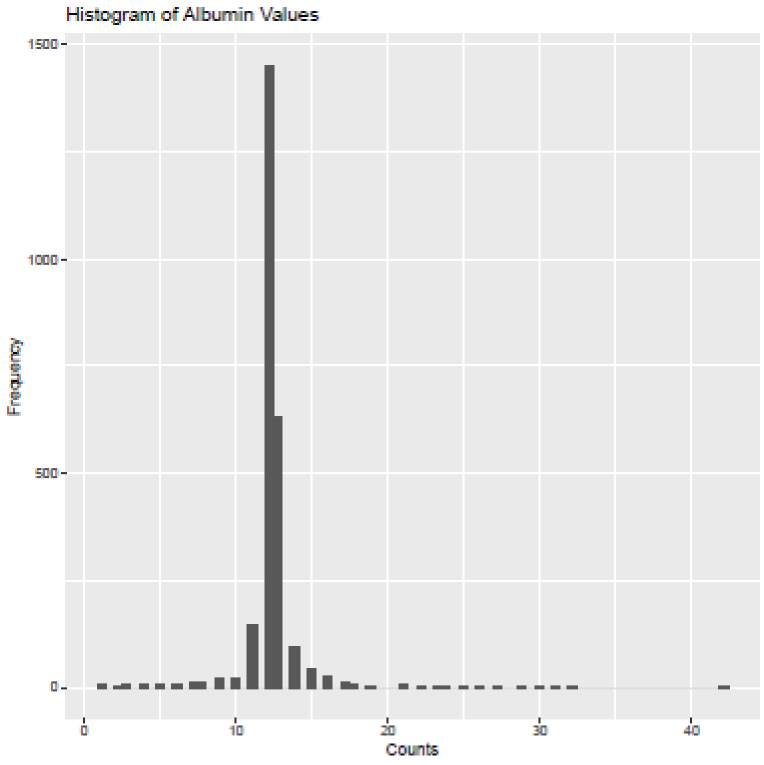

Supplemental data Figure S2.

Histograms of number of counts from which averages were determined during baseline year 1.

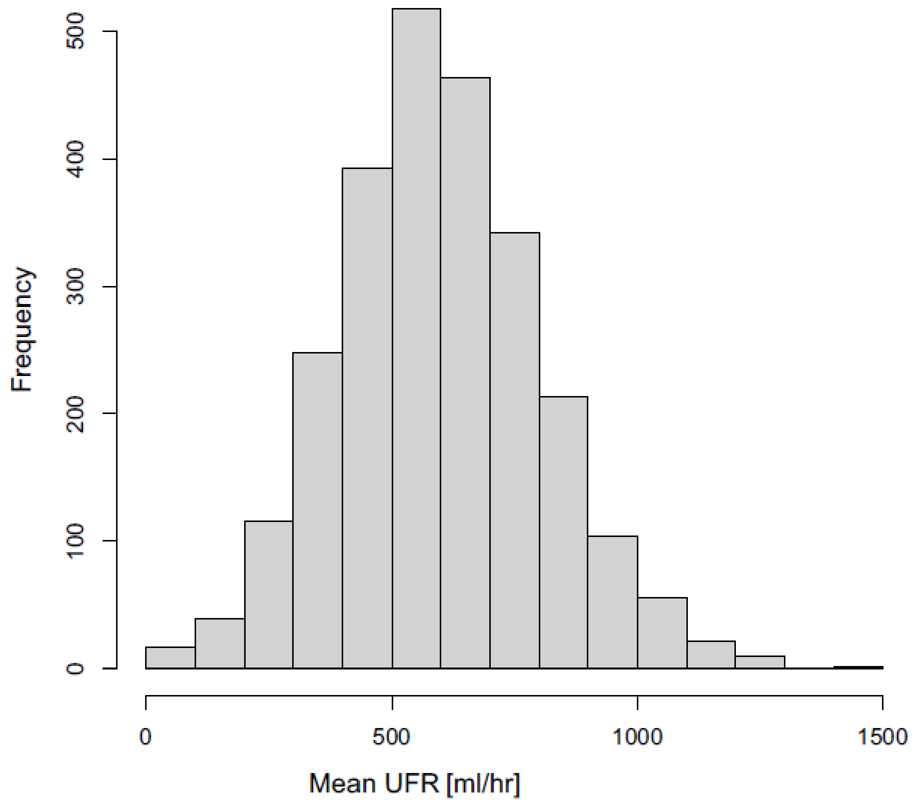

Supplemental data Figure S3a.
Frequency distribution of mean unscaled ultrafiltration rate during the 12-month baseline period (ml/hr).

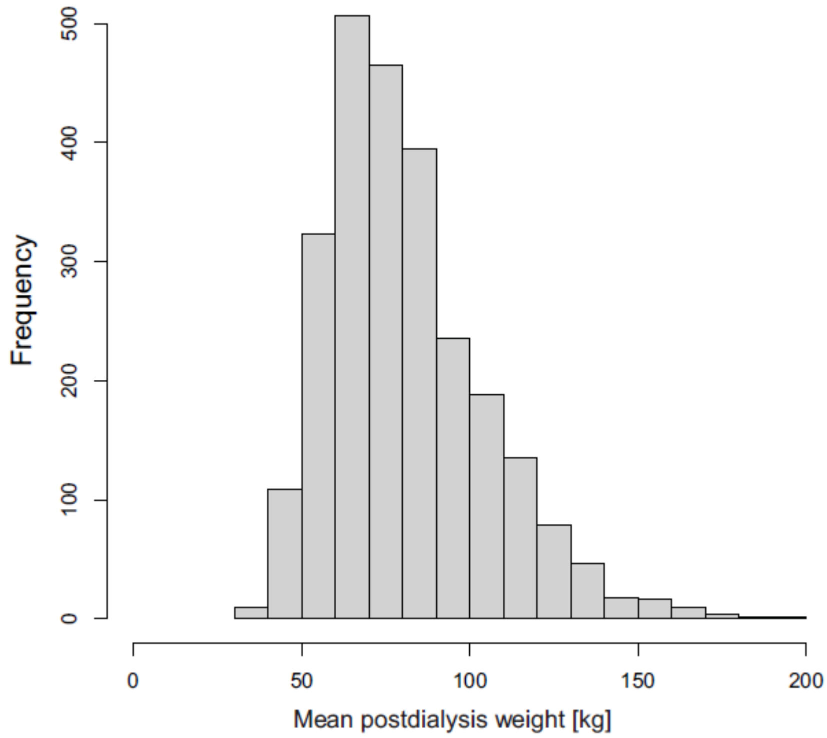

Supplemental data Figure S3b.

Frequency distribution of mean postdialysis weight during the 12-month baseline period (kg).

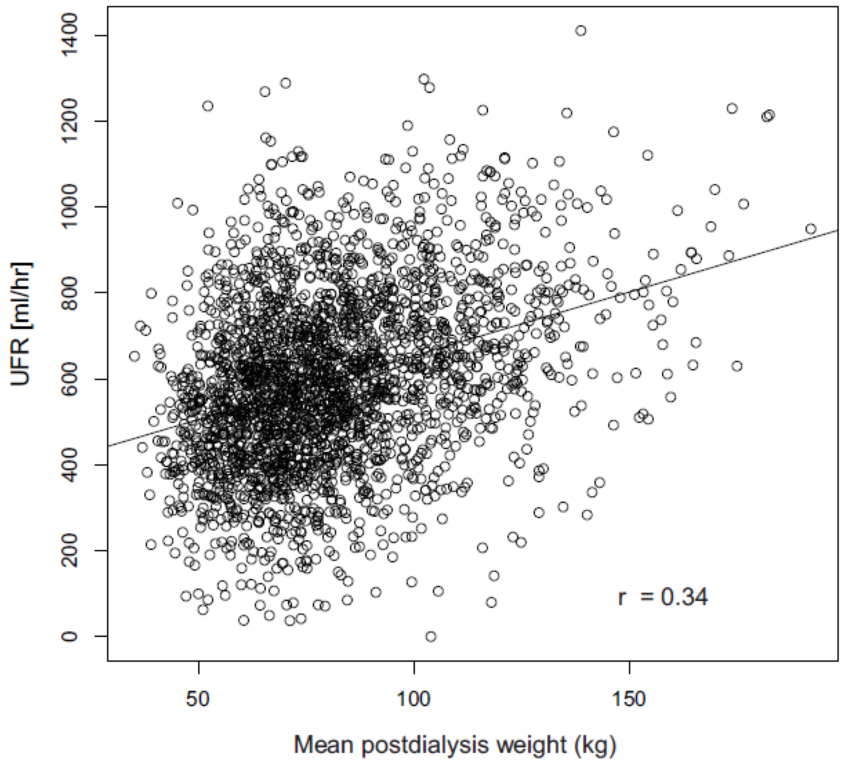

Supplemental data Figure S4a.

Scatterplot of unscaled UFR against postdialysis weight, r = 0.34.

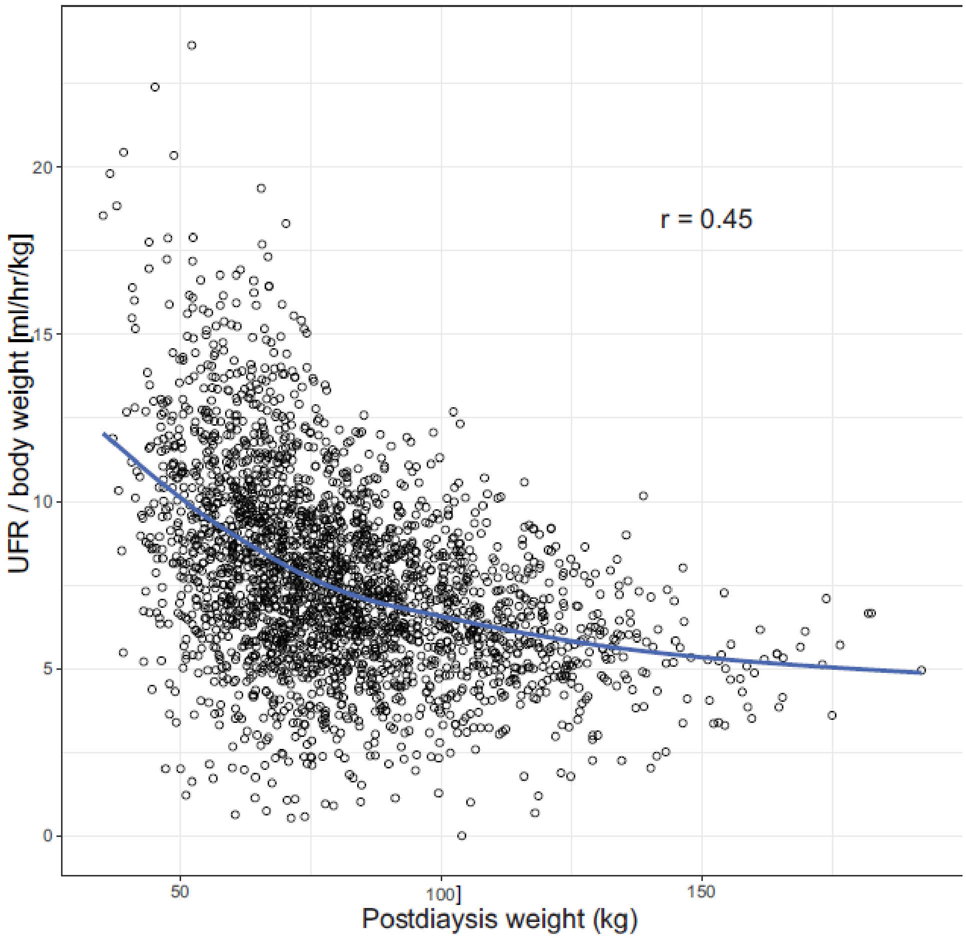

Supplemental data Figure S4b.

Scatterplot of UFR/kg against postdialysis weight, r = 0.45.

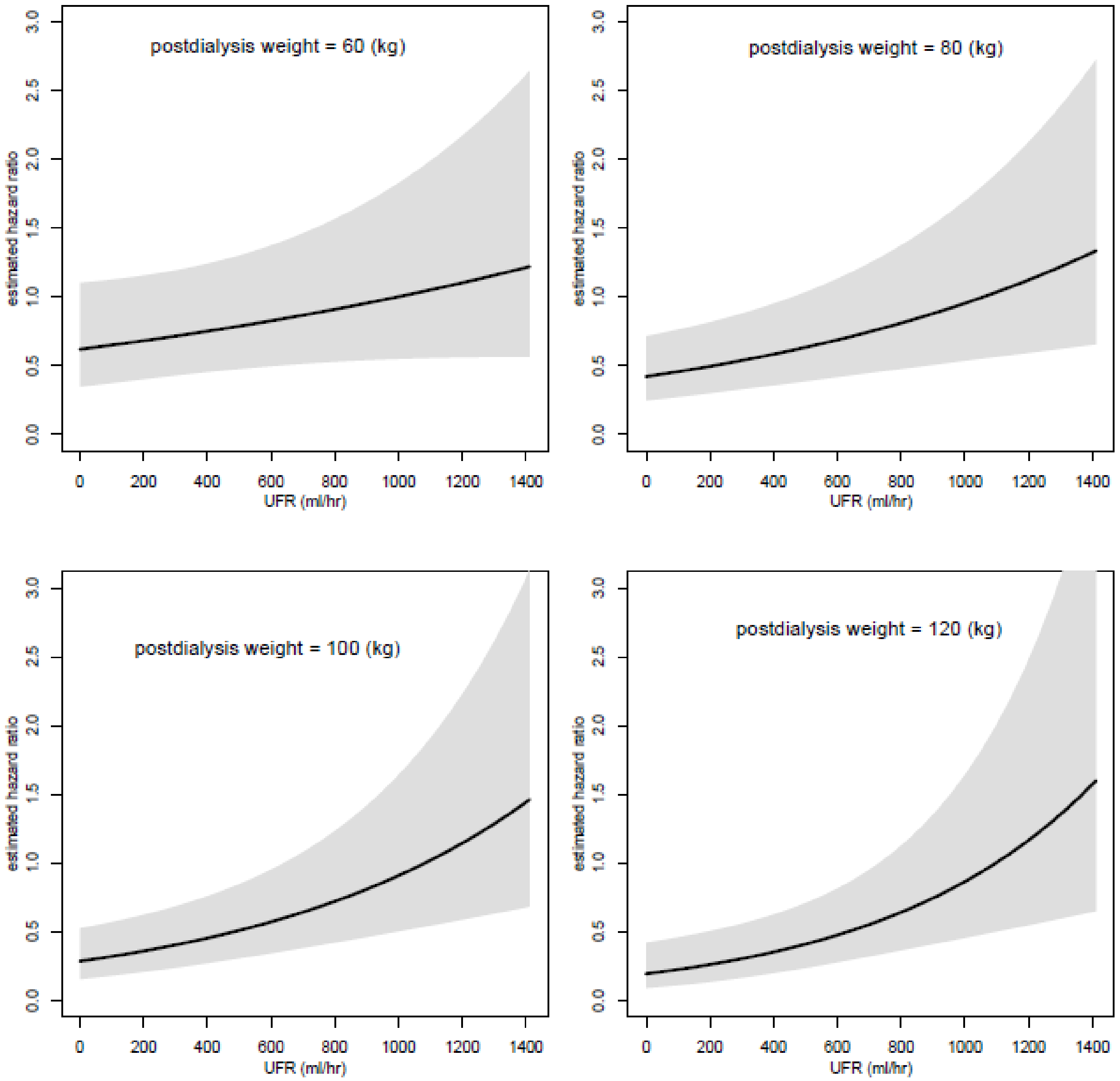

Supplemental data Figure S5. Slice plots showing estimated mortality hazard ratio vs. ultrafiltration rate for various levels of body weight, based on contour plot analysis.

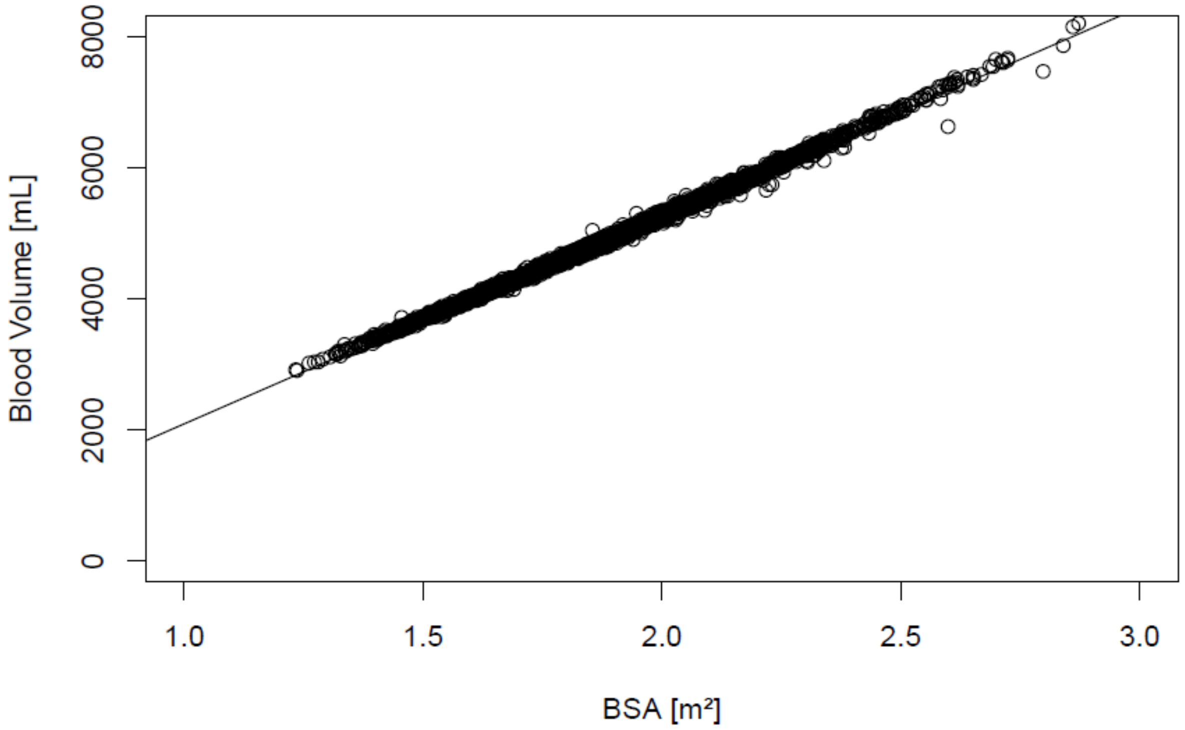

Supplemental data Figure S6.

Scatterplot of estimated blood volume calculated using the Lemmens equation against body surface area.

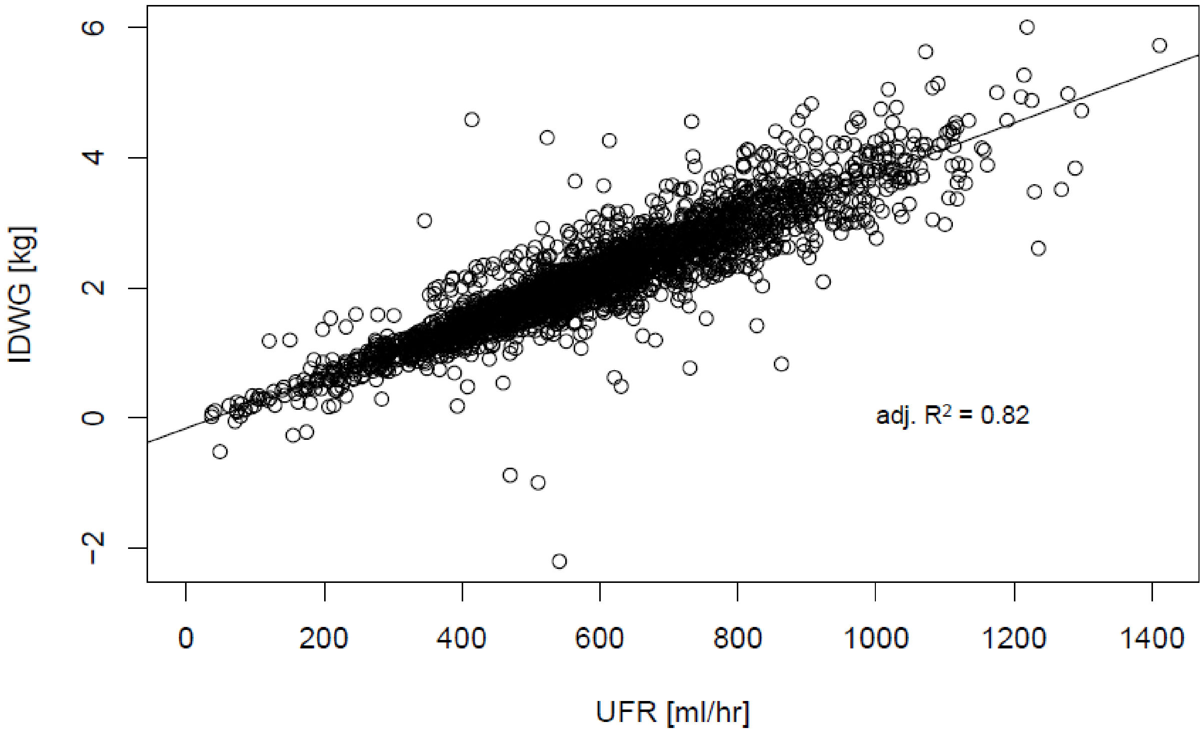

Supplemental data Figure S7.

Scatterplot of average interdialytic weight gain (IDWG) vs. average ultrafiltration rate (UFR) during the baseline period.

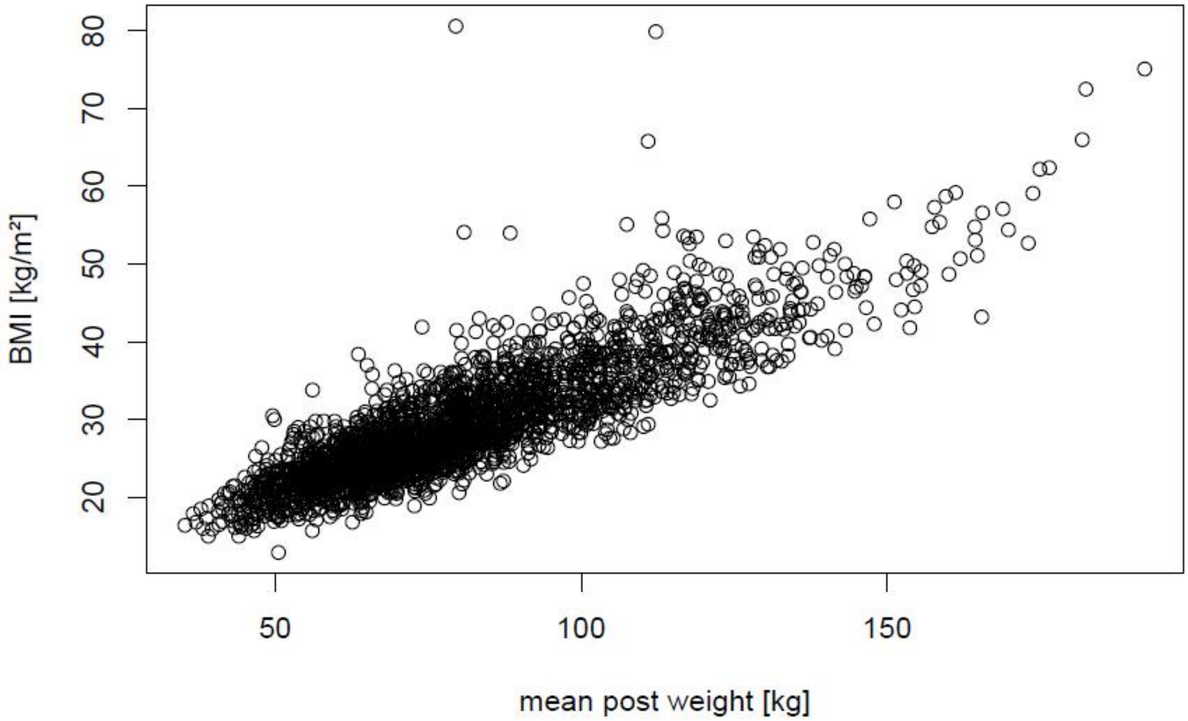

Supplemental data Figure S8.

Scatterplot of postdialysis body weight vs. body mass index.

Supplemental data Table S1. Comparison of UFR averaged over 12 months vs. months 11–12 of year 1.

Data adjusted for race, age, sex, diabetes, predialysis serum phosphorus, albumin, and systolic blood pressure: (MHR = mortality hazard ratio). The contour plot analysis on which the table below is based was set up using the same constraints as in the primary analysis.

**12 month baseline**		**MHR**			
Weight	1	1.1	1.3	1.5	2
50	500	750			
60	530	710	1000		
70	560	700	925	1125	
80	600	700	880	1050	
100	650	725	860	975	1200
120	710	770	875	970	1145
140	760	820	905	985	1125
**2 month baseline**					
Weight					
50	520				
60	545	830			
70	575	750	1280	1400	
80	610	735	970	1200	
100	660	755	900	1040	1300
120	735	800	905	1000	1195
140	800	850	930	1010	1160

STROBE Statement

Topic	Item #	Recommendation	Statement of the authors
**Title and abstract**	1	(*a*) Indicate the study's design with **Title and abstract** as a commonly used term in the title or the abstract.	Study design is stated in *the Abstract,* but not in the *Title.*
		(*b*) Provide in the abstract an informative and balanced summary of what was done and what was found.	A balanced summary of methodology and results is provided in the *Abstract*.
		**Introduction**	
Background/rationale	2	Explain the scientific background and rationale for the investigation being reported.	Background and Rationale are provided in the *Introduction*
Objectives	3	State specific objectives, including any prespecified hypotheses.	Objective and hypotheses are provided in the Thesis Statement at the end of the *Introduction* (**page 3/Line 19 onwards**)
		**Methods**	
Study design	4	Present key elements of study design early in the paper.	Study design is explained in the beginning the *Methods* section (**page 4/Line 2 onwards**)
Setting	5	Describe the setting, locations, and relevant dates, including periods of recruitment, exposure, follow-up, and data collection.	Setting, locations and dates are explained in the beginning the *Methods* section in Study Design (**page 4/Line 2 onwards**). Method of data collection, definition of baseline period and follow-up is provided in the *Methods* in *Study*

			*Design (***page 4/Line 2***).*
Participants	6	(*a*) *Cohort study*—Give the eligibility criteria, and the sources and methods of selection of participants. Describe methods of follow-up.	Patient data selection and data sources are explained in the *Methods* in *Study Design* (**page 4/Line 2 onwards**).
		(*b*) *Cohort study*—For matched studies, give matching criteria and number of exposed and unexposed.	n/a
Variables	7	Clearly define all outcomes, exposures, predictors, potential confounders, and effect modifiers. Give diagnostic criteria, if applicable.	Data sources for all variables are outlined in the *Methods/Measurements* (**page 4/Line 11 onwards**) and confounders that were accounted for are stated in the *Methods/Statistical Analysis* (**page 4/Line 22 onwards**). To evaluate interactions and effect modification between UFR and post-dialysis weight, we present our data on mortality risk in a bivariate fashion as contour plots, a method outlined in *Methods/Statistical Analysis* (**page 4/Line 22 onwards**).
Data sources/ measurement	8	For each variable of interest, give sources of data and details of methods of assessment (measurement). Describe comparability of assessment methods if there is more than one group.	Data sources for all variables are outlined in the *Methods/Measurements* (**page 4/Line 11**).

Bias	9	Describe any efforts to address potential sources of bias.	We conducted a sensitivity analysis restricting baseline to only 2 months (Month 11 and 12) to evaluate potential biases which is outlined in *Methods/Measurements* (**page 4/Line 11 onwards**) and *Methods/Statistical Analysis* (**page 4/Line 22 onwards**).
Study size	10	Explain how the study size was arrived at.	The study size was determined by the available data we had for this particular cohort. This is outlined in the beginning of *Results* **(page 6/Line 1 onwards)** *and the* flowchart of patient inclusion *Supplemental Figure S2*.
Quantitative variables	11	Explain how quantitative variables were handled in the analyses. If applicable, describe which groupings were chosen and why.	This is outlined in *Methods/Measurements* (**page 4/Line 11 onwards**) and *Methods/Statistical Analysis* (**page 4/Line 22 onwards**).
Statistical methods	12	(*a*) Describe all statistical methods, including those used to control for confounding.	All methods are explained in *Methods/Statistical Analysis* **(page 4/Line 22 onwards)**.
		(*b*) Describe any methods used to examine subgroups and interactions.	Interactions were evaluated as outlined in *Methods/Statistical Analysis* **(page 4/Line 22 onwards)**.

		(*c*) Explain how missing data were addressed.	Missing data were excluded from the computation of the mean and the standard deviation. This is commented on in *Methods/Measurements* (**page 4/Line 11 onwards**)
		(*d*) *Cohort study*—If applicable, explain how loss to follow-up was addressed	This is outlined in *Methods/Study Design* (**page 4/Line 2 onwards**).
		(*e*) Describe any sensitivity analyses	We outline the conducted sensitivity analyses in *Methods/Statistical Analysis* **(page 4/Line 22 onwards)**
**Results**			
Participants	13	(a) Report numbers of individuals at each stage of study— e.g. numbers potentially eligible, examined for eligibility, confirmed eligible, included in the study, completing follow-up, and analyzed.	Numbers are reported in the beginning of *Results* **(page 6/Line 1 onwards)** *and the* flowchart of patient inclusion *Supplemental Figure S2*.
		(b) Give reasons for non-participation at each stage	This is commented on in *Methods/Measurements* (**page 4/Line 11 onwards**) *and the* flowchart of patient inclusion *Supplemental Figure S2*.
		(c) Consider use of a flow diagram	A flowchart is included as *Supplemental Figure S2*.

Descriptive data	14	(a) Give characteristics of study participants (e.g. demographic, clinical, and social) and information on exposures and potential confounders.	This is provided in *Table 1*.
		(b) Indicate number of participants with missing data for each variable of interest.	This is reported in the flowchart of patient inclusion *Supplemental Figure S2*.
		(c) *Cohort study*—Summarize follow-up time (e.g. average and total amount)	This is outlined in *Methods/Study Design* (**page 4/Line 2 onwards**).
Outcome data	15	*Cohort study*—Report numbers of outcome events or summary measures over time	This is reported in *Results/Survival Analysis* (**page 6/Line 14 onwards**).
Main results	16	(*a*) Give unadjusted estimates and, if applicable, confounder-adjusted estimates and their precision (eg, 95% confidence interval). Make clear which confounders were adjusted for and why they were included.	This is reported in *Results/Survival Analysis* (**page 6/Line 14 onwards**).
		(*b*) Report category boundaries when continuous variables were categorized.	n/a

		(*c*) If relevant, consider translating estimates of relative risk into absolute risk for a meaningful time period.	n/a; not relevant for the current analysis.
Other analyses	17	Report other analyses done - e.g. analyses of subgroups and interactions, and sensitivity analyses.	All analyses are reported in the *Results* (**page 5/Line 2 onwards**); sensitivity analyses in *Results/Sensitivity Analyses* (**page 5/Line 35 onwards**)
**Discussion**			
Key results	18	Summarise key results with reference to study objectives.	Key results are outlined at the beginning of the *Discussion* (**page 7/Line 13 onwards**).
Limitations	19	Discuss limitations of the study, taking into account sources of potential bias or imprecision. Discuss both, direction and magnitude of any potential bias.	Strengths and Limitations are outlined in the *Discussion* (**page 8/Line 10 onwards**).
Interpretation	20	Give a cautious overall interpretation of results considering objectives, limitations, multiplicity of analyses, results from similar studies, and other relevant evidence	The current results are discussed and interpreted in the context of the currently published literature in *Discussion* (**page 7/Line 10 onwards**).
Generalizability	21	Discuss the generalizability (external validity) of the study results.	External validity is discussed in the strengths and limitations are outlined in the *Discussion*

			(**page 8/Line 10 onwards**).
**Other information**			
Funding	22	Give the source of funding and the role of the funders for the present study and, if applicable, for the original study on which the present article is based.	No external funding was received and this is stated on the *Cover Page*.
